# *In vivo* biocompatibility analysis of the recellularized canine tracheal scaffolds with canine epithelial and endothelial progenitor cells

**DOI:** 10.1080/21655979.2021.2020392

**Published:** 2022-02-02

**Authors:** Gustavo de Sá Schiavo Matias, Ana Claudia O. Carreira, Vitória Frias Batista, Hianka Jasmyne Costa de Carvalho, Maria Angelica Miglino, Paula Fratini

**Affiliations:** a Department of Surgery, School of Veterinary Medicine and Animal Science, University of São Paulo, São Paulo, Brazil; b Neuromuscular Disease Laboratory, Faculdade de Medicina do ABC (FMABC), Santo André, Brazil

**Keywords:** Cartilage, transplants, tracheal collapse, tissue engineering, decellularized scaffolds

## Abstract

Decellularized extracellular matrix (ECM) has frequently been applied as a biomaterial for tissue engineering purposes. When implanted, their role can be essential for partial trachea replacement in patients that require a viable transplant solution. Acellular canine tracheal scaffolds with preserved ECM structure, flexibility, and proteins were obtained by high pressure vacuum decellularization. Here, we aimed to evaluate the cell adhesion and proliferation of canine tracheal epithelial cells (EpC) and canine yolk sac endothelial progenitor cells (YS) cultivated on canine decellularized tracheal scaffolds and test the in vivo biocompatibility of these recellularized scaffolds implanted in BALB-c nude mice. In order to evaluate the recellularization efficiency, scaffolds were evaluated by scanning electron microscopy (SEM), immunofluorescence, DNA quantification, mycoplasma test, and in vivo biocompatibility. The scaffolds sterility was confirmed, and EpC and YS cells were cultured by 7 and 14 days. We demonstrated by SEM, immunofluorescence, and genomic DNA analyzes cell adhesion to tracheal ECM. Then, recellularized scaffolds were in vivo subcutaneously implanted in mice and after 45 days, the fragments were collected and analyzed by Hematoxylin-Eosin and Gömori Trichrome staining and PCNA, CD4, CD8, and CD68 immunohistochemistry. In vivo results confirmed that the implanted tissue remains preserved and proliferative, and no fibrotic tissue process was observed in animals. Finally, our results showed the recellularization success due the preserved ECM proteins, and that these may be suitable to future preclinical studies applications for partial trachea replacement in tissue engineering.

## 1. Introduction

Tissue engineering techniques have been achieve great potential in the tissues and organs functional restoration and, therefore, can provide as a promising approach in tracheal reconstruction and other tissues [1,2]. However, in cases of tracheal dysfunction, such as the case of patients with long-segment abnormalities, tracheal stenosis or collapse, it is considered to be one of the most challenging for investigation. Additionally, one of the restrictions is the maximum resection length for replacement up to 6 cm [3]. Although tracheal transplantation is possible, its clinical applications are limited due to the need for an immune suppression lifelong use. Furthermore, the respiratory and secretory function of the trachea, together with its segmental blood supply, hinders the regeneration of this organ [4].

However, the option of the ideal scaffold in which the cells will be seeded is as important as the source of stem cells in tissue engineering [5–7]. The airways repair clearly define that the repair of the using artificial prostheses is extremely difficult, since for the repair it is essential to mediate the physiology of tracheal movement (breathing, swallowing, and coughing), additionally to the external environmental conditions that can interfere with bacterial contamination preventing local healing and its optimal functioning after transplantation [8,9]. Furthermore, an ideal tracheal scaffold should exhibit a tubular shape similar to the natural trachea, porosity for cell growth, and biocompatibility [10].

Currently, the use of decellularized scaffolds for cell repopulation is a promising tool in tracheal tissue engineering aiming at its replacement [7,9,11–14]. The use of decellularized scaffolds reveals that it is possible to have a non-immunogenic ECM, which would not cause rejection problems. Also, these matrices provide a flexible three-dimensional structure, and ECM properties that helps in cell migration, differentiation, and migration [11].

Recently studies demonstrate the decellularized tracheal scaffolds efficiency in anchoring different cell lines in the recellularization process with tracheal cells [15,16], human embryonic kidney cells [17] and mouse-induced stem cells (iPS) [18]. Nevertheless, factors such as tissue cells and vascular cells in the recellularization of tracheal scaffolds must be considered for ideal tissue functionality [4,19].

Therefore, we herein intended to evaluate the cell adhesion and proliferation of EpC and YS cells cultivated on canine decellularized tracheal scaffolds targeting verify if these cell types are able to adhere on decellularized tracheal scaffolds, hypothesized that these recellularized scaffolds could be applied for future applications for partial replacement of the trachea and test the immune response in vivo of these recellularized scaffolds implanted in BALB-c nude mice.

## 2. Material and methods

### 2.1. Trachea decellularization validation

Briefly, the tracheal segments (n = 24 rings) were thrice washed with 1% ethylenediamine tetraacetic acid (EDTA) solution (#197072, Synth) 5 minutes each to blood removal. Then, the canine trachea segments were washed thrice in phosphate-saline buffer ([PBS] 136.9 mM NaCl, 26, 8 mM KCl, 14.7 mM KH2PO4 and 8 mM Na2HPO4, pH 7.2, LGC Bio) under stirring (60 rpm) at room temperature for 15 minutes each to removal of contaminants from tissue. After, they were immersed for 8 days (changed twice daily) in 4% sodium dodecyl sulfate ([SDS] – LGC Biotechnology, #13-1313-01) under orbital agitation (60 rpm) at room temperature to remove the cells. Following, trachea scaffolds were 2-days extra stored in high pressure vacuum chamber (<1000 Pa) for 2 hours each immersed in 4% SDS for complete cell removing. Finally, were washed in PBS for 1 hour. All solutions were supplemented with 0.5% antibiotic (ATB) Penicillin/Streptomycin (LGC Biotechnology, #BR30110-01) to sterilize the scaffolds. Next, decellularized samples from canine trachea were genomic DNA quantified, PFA fixed and stained by Hematoxylin-Eosin (H&E), Alcian Blue (EasyPath, HistoKit) and 4,6-diamidino-2-phenylindole fluorescence (DAPi) as described by [20].

### 2.2. Canine tracheal epithelial cells (EpC) isolation/yolk sac endothelial progenitor cell culture (YS)

Two fresh tracheas were used for cell isolation. Samples were washed with 1X PBS + 0.5% ATB and transferred to a petri dish for tissue digestion (collagenase, Type I, 1 mg/m, #17018029, ThermoFisher) for 1 hour and a half at 37°C. Following, they were centrifuged (1200 rpm, clinical centrifuge) and culture medium (α-MEM, LGC Biotechnology) was added, supplemented with 10% fetal bovine serum (FBS) and 1% ATB, and incubated at 37°C in a humid atmosphere. YS cells were previously described by the group [21,22]. Both lineage were cultured in 35 mm (*Corning*) petri dish with α-MEM medium at same conditions as above mentioned. At 80% confluence, cells were trypsinized (0,25%, LGC Bio) for subsequent freezing in complete medium (FBS) containing dimethylsulfoxide (10% DMSO, (CH₃)₂SO, LGC Bio) and preserved in liquid nitrogen (NL_2_).

### 2.3. Canine tracheal epithelial cells (EpC) immunofluorescence/recellularized scaffolds validation

EpC cells were viable after unfreezing and were seeded on coverslips in a 24-well plate (5 × 10^4^ cells) for 7 days. Cells were PFA (Synth) fixed for 20 minutes. Coverslips were washed in PBS 1% + 0.5% Tween-20 (Synth) solution and incubated with primary antibodies diluted in 2% PBS + BSA: E-Cadherin (24E10, Cell Signaling, 1:200), N-Cadherin (13A9, Cell Signaling, 1:200), Vimentin (GTX35160, GeneTex, 1:100), Cytokeratin 18 (RGE53, Novus, 1:200), β2 Tubulin (sc-47751, Santa Cruz Biotechnology, 1:200), VEGF (ac12013315, Bioss), CD44 (#GTX80086,Genetex,1:100), TGF-β (#SC-146, Santa Cruz Biotechnology, 1:100), CD31 (#ab32457, Abcam, 1;100), PCNA (ma5-11358, Invitrogen, 1:100) and hyaluronic acid (#c41975, LS Bio, 1:100) for 1 hour at 37°C. Then, Alexafluor 488/594 secondary antibodies (#A30008/#A-11094, Thermo Fisher) were added at concentration 1:200 for 1 hour at 4°C. Recellularized scaffolds and the coverslips were carefully washed, followed by DAPi (Sigma-Aldrich) incubation for 10 minutes for nuclei stain. The samples were analyzed in Confocal Microscope – Olympus Fluo View 1000 (CADI-FMVZ).

### 2.4. Canine trachea scaffolds recellularization/DNA quantification

Decellularized canine trachea scaffolds fragments (5 × 5 mm) were cultured for recellularization with a quantity of 5 × 10^4^ EpC and YS cells for 7 days and 14 days in static cell culture. Cells lineages were seeded in non-adherent plates (*Sarstedt*), and the amount of genomic DNA of recellularized scaffolds was carried out. Briefly, gDNA was isolated from 50 mg dry tracheal scaffolds fragments (control, decellularized, or recellularized) using IllustraTM Tissue and Cells Genomic Prep Mini Spin Kit (GE Healthcare), according to the protocol established by the group [22]. Samples were digested with Proteinase K and lysis buffer at 56°C for 2 h. They were analyzed in a spectrophotometer at 260 nm (Nanodrop; Thermo). The data was presented as gDNA total (ng)/tissue (mg). To verify the ECM architecture and cell adhesion, scanning electron microscopy (SEM) analysis was performed as described by [23].

### 2.5. Statistical analysis

One-way ANOVA followed by Dunnett’s multiple comparisons test was performed using GraphPad Prism version 8.0.0 for Windows, GraphPad Software, San Diego, California USA, www.graphpad.com”. A p-value less than 0.05 was considered statistically significant.

### 2.6. PCR test for mycoplasma detection

Eleven species of Mycoplasma *(M.fermentans, M. hyorhinis, M. arginini, M. orale, M. salivarium, M. hominis, M. pulmonis, M. arthritidis, M. neurolyticum, M. hyopneumoniae, M. capricolum)* and one of Ureaplasma was carry out in order to detect potential presence of contamination in the decellularized canine trachea scaffolds and recellularized scaffold with EpC and YS cells. Adapted from *[24]* using the polymerase chain reaction (PCR) technique (step PCR1), followed by the Nested-PCR technique (PCR2) and analysis of the DNA fragments on agarose gel stained with ethidium bromide

### 2.7. In vivo biocompatibility test of canine tracheal scaffolds

Six male BALB-c nude mice (4–6 weeks) were acquired from the Central Animal Facility of the Faculty of Medicine of the University of São Paulo (FMUSP) and kept in the Animal Reproduction Department from FMVZ-USP. Decellularized and recellularized tracheal scaffolds (5 × 5 mm) were individually implanted subcutaneously into the right and left medial dorsal thoracic region (1 cm incision). Two animals were subjected to experimental group analysis: **Control group**: Right side – decellularized scaffold; Left side – sham, only incision (without biomaterial); **Recellularized scaffolds group (7 days**): Right side – scaffold with EpC cells; Left side – scaffold with YS cells; **Recellularized scaffolds group (14 days**): Right side – scaffold with EpC cells; Left side – scaffold with YS cells. Then, animals were submitted to anesthetic induction, placed individually inside an induction chamber, which received isoflurane as an inhaled anesthetic agent at the appropriate concentration with oxygen gas: Induction Phase 3–4% isoflurane; Maintenance Phase 1–2%. Anesthesia lasted about 5–10 minutes; time required for scaffolds implantation on both animals sides. Finally, the animal received pure oxygen for approximately 5 minutes to avoid hypoxia. No immunosuppressive medication was administered during the experiment. After implant scaffolds surgery, analgesia was performed using the opioid Tramadol at a dose of 20 mg/Kg (IM) diluted 2.5 times in ringer lactate, and the animal did not present pain (12 to 24 hours) after surgery. The animals were placed in numbered cages (1 animal/cage) containing sterile wood shavings, environmental enrichment, in appropriate rooms with controlled temperature between 20°C and 22°C, with a 12-hour light/dark cycle. The experiment lasted 45 days. After this period, the animal was euthanized using an anesthetic overdose of Xylazine 30 mg/Kg (I.P) and Ketamine 300 mg/Kg (I.P). Euthanasia was checked due to the absence of heart and respiratory rate. The implanted scaffolds regions were photodocumented and collected, Tissue-Tek OCT included (#4583-1, Sakura – Torrance, USA) and kept in a freezer at −80°C for 10 minutes. Subsequently, the materials were sectioned (10 µm) in a cryostat (Leica CM1520®), adhered to silanized starfrost sheets (*Knittel Glass*), and submitted to histological stains (H&E, Gömori Trichrome and immunohistochemistry).

### 2.8. In vivo immunohistochemistry validation

*In vivo* implanted samples in the subcutaneous tissue of mice were collected and analyzed by immunohistochemistry using the Dako EnVisionTM FLEX Detection System, High pH Link (#K8000) according to the manufacturer’s instructions. The slides were washed in a buffer solution and the sections surrounded with a hydrophobic pen. For ECM analysis, the primary antibodies diluted in PBS + 2% bovine serum albumin (BSA, #A1310-05, LGC Biotechnology) were used: PCNA (ma5-11358, Invitrogen, 1:100), CD68 (ab9555, abcam, 1:200), CD4 (PA0427, Leica, 1:200), CD8 (PA0183, Leica, 1:200). Following incubation time, the slides were washed, and HRP solution were incubated for 30 minutes in a humid chamber. Subsequently, the sections revels was performed using a reaction with diaminobenzidine (DAB, 30 μl per section: 1 ml of substrate + 1 drop of chromogen). Slides were counterstained with Harris Hematoxylin for 40 seconds, washed with H_2_O distilled and rehydrated. The samples were photodocumented in a Nikon Eclipse 80i microscope.

## 3. Results

First, aiming to achieve our goals, canine tracheal scaffolds applied to the combined decellularization process was carried out by the detergent action SDS and high vacuum pressure showed reduced values of genomic DNA (13,5 ± 1,1ng/mg [SD]) when compared to the control tissue (115.38 ± 57,91ng/mg [SD]). Additionally, the scaffolds maintained a preserved native structure after the cell removal process. By H&E staining and DAPi fluorescence (Suppl Figure S1a-b; e-f), it was possible to verify the cell nuclei removal of tracheal cartilage regions. Furthermore, when analyzed by Alcian Blue staining (Suppl Figure S1(c–g)), it was observed that the main structuring proteins of the tracheal ECM, such as glycosaminoglycans (GAGs) preserved after the decellularization process. Additionally, by SEM, the ultrastructural analysis of the decellularized tracheal ECM reveals the three-dimensional tissue structure preserved after cell removal process when compared to the control tissue (Suppl Figure S1(d–h)).

Morphologically, EpC cells presented an elongated morphology, with regular cytoplasmic membrane, small and circular nuclei (Suppl Figure S2(a)). Epithelial cells grow up progressively and were unchanged after trypsinization and cell freezing. Cultured with decellularized scaffolds, they showed cell/ECM interaction, and in 14 days, the cells showed better proliferation when compared to the 7-day culture (Suppl Figure S2(b,c)). The morphology of YS cell was predominantly fibroblastoid with elongated, fusiform, and reduced cytoplasm (Suppl Figure S2(d)). These, when cultivated with scaffolds for 7 and 14 days, showed an increase in cell proliferation (Suppl Figure S2(f)).

By EpC cells immunocytochemical characterization, it was possible to verify the expression of epithelial markers that make up the intermediate filaments of respiratory tract ciliated cells: β-Tubulin (Suppl Figure S3(A^I^-A^III)^)) present in nuclei and intermediate filaments and CK18 and Phalloidin (Suppl Figure S3A^I^,B^I^-B^III^/H^I^-H^III^) in the cellular intermediate filament. E-Cadherin positive expression (Suppl Figure S3(A^I^,C^I^-C^III^)) and N-Cadherin (Suppl Figure S3A^I^,D^I^-D^III^) in the intermediate filaments of epithelial cells can provide efficiency in mediating cell/cell adhesion. Immunofluorescence of the mesenchymal cell marker Vimentin (Suppl Figure S3(A^I^,E^I^-E^III^)) in epithelial cells can confer the epithelial cells plasticity for cell differentiation. Epithelial cells were positive for the VEGF marker (Suppl Figure S3(A^I^,G^I^-G^III^)) that aid in cell migration, vasculogenesis, endothelial cell growth, and proliferative potential observed by positive PCNA marker immunofluorescence (Suppl Figure S3(A^I^,F^I^-F^III^)).

**Figure 1.**
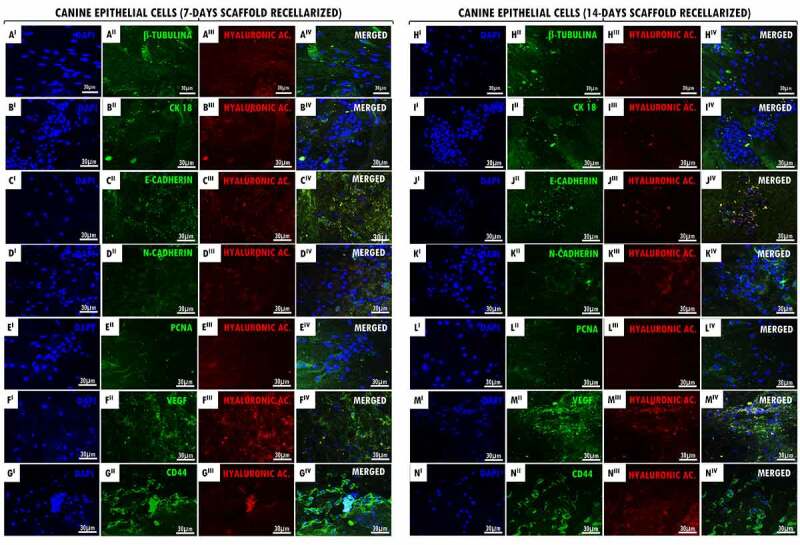
Recellularized canine trachea scaffolds immunofluorescence with EpC cells (7 and 14 days). In (A^I^ – N^I^), DAPI immunostaining (blue) for canine tracheal epithelial cell nuclei. In (A^II^ – N^II^), immunostaining (green) for antibodies: β-Tubulin, CK18, E-Cadherin, N-Cadherin, PCNA, VEGF, CD44, respectively. In (A^III^ – N^III^), hyaluronic acid immunostaining (red) for the canine trachea ECM. In (A^IV^- N^IV^), merged. Scale bar: 30 µm.

**Figure 2.**
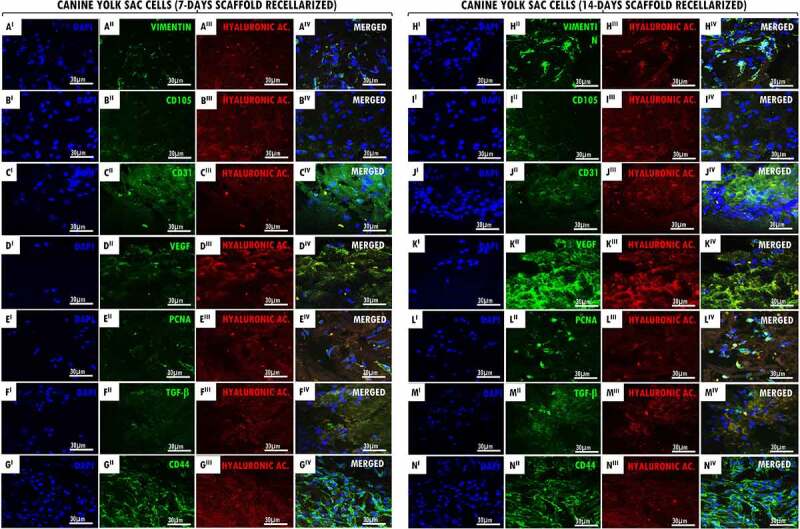
Recellularized canine trachea scaffolds immunofluorescence with YS cells (7 and 14 days). In (A^I^ – N^I^), DAPI immunostaining (blue) for canine yolk sac endothelial progenitor cell nuclei. In (A^II^ – N^II^), immunostaining (green) for antibodies: vimentin, CD105, CD31, VEGF, PCNA, TGF-β and CD44, respectively. In (A^III^ – N^III^), hyaluronic acid immunostaining (red) for the canine trachea ECM. In (A^IV^- N^IV^), merged. Scale bar: 30 µm.

**Figure 3.**
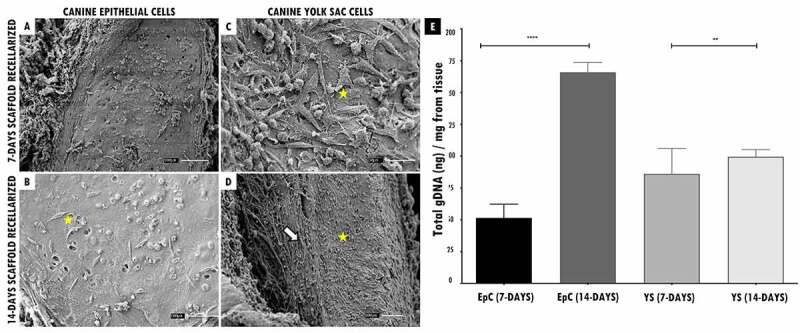
Genomic DNA quantify and SEM of recellularized tracheal scaffolds (7 and 14 days) with EpC and YS cells. In (a-b) canine trachea recellularized with EpC (7 and 14 days of in vitro culture) respectively. In (c-d), canine trachea recellularized with YS (7 and 14 days of in vitro culture). Observe the adhesion of cell types in the hyaline cartilage region of the trachea (star). Preserved organ structure, allowing cell/ECM interaction. In the perichondrium region (empty arrow), it is possible to have cells anchored in the collagen fibers of the tissue. (e) N = 3. Average ± DS. Anova ****p < 0,0001; ** p < 0,01. Scale bar: (A, B and D: 100 µm) (C: 30 µm).

Then, in order to prove the recellularization process efficiency, it was collected the α-MEM culture medium in contact with the decellularized scaffolds and compared with the control medium. All samples tested were negative for the 11 different mycoplasma species and one of ureaplasma, as observed in the agarose gels (PCR 1 and 2), demonstrating effectiveness of decellularized scaffolds sterility in cell culture (Suppl. Figure S4). Next, recellularized scaffolds (7 and 14 days) were collected for immunofluorescence analysis (Figures 1 and 2), genomic DNA quantification and ultrastructural SEM analysis (Figure 3) aiming to verify the cell/ECM adhesion and proliferation at decellularized tracheal scaffolds.

It was demonstrated by immunofluorescence that the cell proliferation and adhesion of EpC and YS cells in both cultured period (7 and 14 days). Our results demonstrated that the positive expression of hyaluronic acid (GAG) supported the interaction tracheal ECM and cells (Figures 1 and 2(A III,N III)). In recellularized scaffolds at 7 and 14 days with EpC and YS cells, we demonstrated the CD44 glycoprotein-positive expression on the cell surface, demonstrating cell–cell interactions, cell adhesion, and migration in the ECM (Figures 1 and 2(G II,N II)). The positive expression of VEGF in EpC cells adhered to scaffolds demonstrates that they possibly have the signaling ability to stimulate the formation of blood vessels (Figure 1(D II,K II)). Also, this can be observed in recellularized scaffolds with YS cells; however, they also have the character of angiogenesis (Figure 2(D ^II^,K^II^)). Proliferative cell nuclear antigen (PCNA) positive expression demonstrated the proliferative cells activity when associated with decellularized matrix (Figures 1 and 2(E^II^,L^II^)), as well as the cell proliferation demonstrated by the transforming growth factor beta (TGF-β) expression in YS cells (Figure 2(F^II^,M^II^)). It was observed the positive expression of antibodies in the intermediate filaments of epithelial cells (β-Tubulin, CK18, E-Cadherin e N-Cadherin) in the 7- and 14-day recellularized scaffolds with EpC cells (Figure 1(A^II^-D^II^,H^II^-K^II^)), respectively. Vimentin immunoexpression in the intermediate filaments of YS cells (Figure 2(A^II^,H^II^)), as well as the CD105 positive expression that acts as a TGF receptor (Figure 2(B II,III)), and CD31 (Figure 2(C^II^,J^II^)) demonstrate that they induce cell activation and proliferation when adhered to tracheal ECM.

Recellularized scaffolds were ultrastructurally analyzed by SEM revealing the collagen fibers were preserved, allowing the EpC (Figure 3(a,b)) and YS (Figure 3(c,d)) cells to adhere to the connective basal lamina and the hyaline cartilage region of the tracheal ECM at 7 and 14 days, additionally to cellular morphology preservation. Fairly, YS recellularized scaffolds demonstrated better adhesion in the cartilage regions, as well as in the collagen fibers present at the extremities of the perichondrium layer.

The genomic DNA quantifying from recellularized canine tracheal scaffolds for 7 and 14 days (Figure 3(e)), it was demonstrating a significant increase of EpC and YS cells after recellularization period when compared to the values obtained from decellularized scaffolds (13.5ng/mg of gDNA). The 14 days decellularized scaffolds revealed a greater cell adhesion to tracheal ECM. Recellularized tracheas with EpC cells, for a period of 7 days, showed values of 51.46 ng/mg (sd = 10.80), while at 14 days in vitro, they showed genomic concentration of 165,9 ng/mg (sd = 7.82). On the other hand, recellularized tracheas with YS cells, showed values of 86.06 ng/mg (sd = 19.96) at 7 days, while at 14 days in vitro, they showed a concentration of 99.51 ng/mg (sd = 5.5).

**Figure 4.**
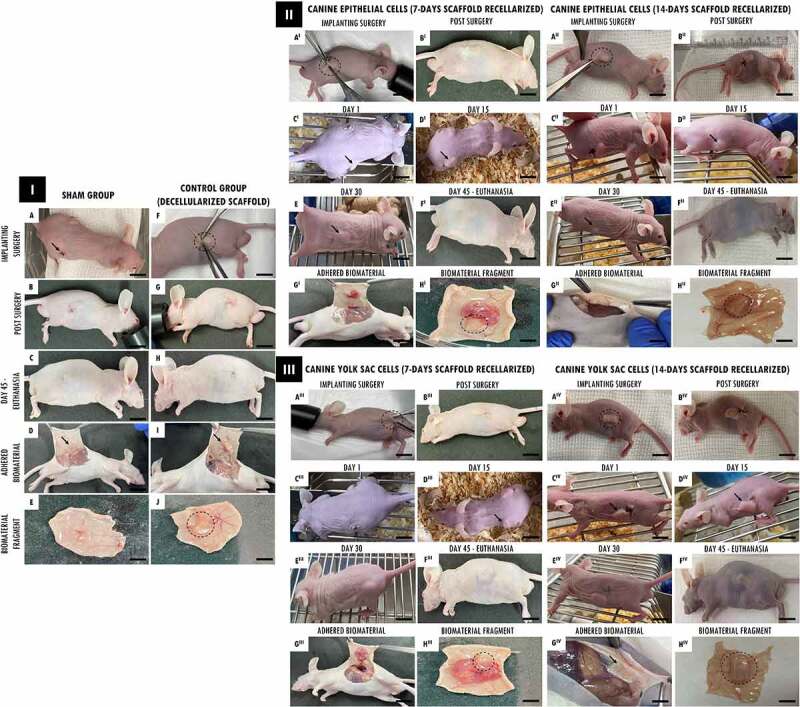
*In vivo* biocompatibility test of tracheal scaffolds implanted in the subcutaneous tissue of balb-C nude mice. I. In (a) surgical incision (1 cm) in the control animal of the sham group (without scaffold) and implantation of the decellularized scaffold (circle) in the control animal (f). In (b-c, g-h), post-surgical animals monitoring. In (d, i) no macroscopic changes observed in the regions. In (e) skin fragment, interne face, epidermis with a slightly translucent aspect, with a slightly elevated focal area with slightly reddish. In (j) Presence of decellularized scaffold adhered to the dermis, with an elevated surface, surrounded by discrete adjacent connective tissue, and presence of vessels in adjacent tissue. II and III. In (A^I^, A^I^, A^III^, A^IV I^) surgical incision (1 cm) in the animals implantation of scaffolds recellularized from 7 and 14 days with EpC (circle) and YS cells, respectively. In (B^I^-E^I^, B^II^-E^II^, B^III^-E^III^, B^I^-E^IV^), post-surgical animals monitoring. In (C^I^ and D^I^, C^II^ and D^II^, C^III^ and D^III^, C^IV^ and D^IV^) observe discrete edema in the implant region (arrow). In (E^I^, E^II^,E^III^, E^IV^) implant region with reduced edema. In (G^I^,G^II^,G^III^,G^IV^) Flat skin, inner surface, deep dermis with moderately translucent appearance. Presence of recellularized scaffold adhered to the dermis, with an elevated surface, surrounded by reddish colored adjacent tissue, with a soft consistency and presence of vessels in adjacent tissue. (H^I,^ H^II^, H^III^,H^IV^) Skin with recellularized scaffold fragment adhered to the dermis (circle), with an elevated surface, surrounded by a discrete layer of connective tissue. Presence of vessels in adjacent tissue, slightly reddish. Scale bar: 1 cm.

Followingthis , the recellularized scaffolds were tested by the in vivo biocompatibility test, and all animals survived the experimental period. Macroscopically, the implanted scaffolds persisted in the same location, with no noticeable shrinkage or structure and flexibility trachea changes. (Figures 4 I(a,f), 4 II(A I-B I,A II-B II), 4 III(A III-B III,A^IV^-B^IV^)). After 45 days of experimental, when euthanasia was performed. The scaffolds revealed normal characteristics of stiffness, reddish appearance, and adjacent vessels formation presence in the implant region, demonstrating that the tissue remained alive, without tissue necrosis appearance (Figures 4 I(e,j), 4 II(G I,H I,G II -H II), 4 III(G^III^-H^III^,G^IV^-H^IV^)).

The animals that received the 7-day recellularized scaffolds with EpC and YS cells showed discreet edema in the implanted region, with a subsequent decrease after the 15th day (Figure 4 (II-D^I^,III-D^III^)). Otherwise, the animals that received 14-day recellularized scaffolds with EpC and YS cells showed mild edema in the implant region, with no apparent tissue accumulation at the site. Still, no edema, inflammation, or fibrotic process were observed in the animals implanted with decellularized scaffolds and sham group (Figure 4 I(d,e,i)).

**Figure 5.**
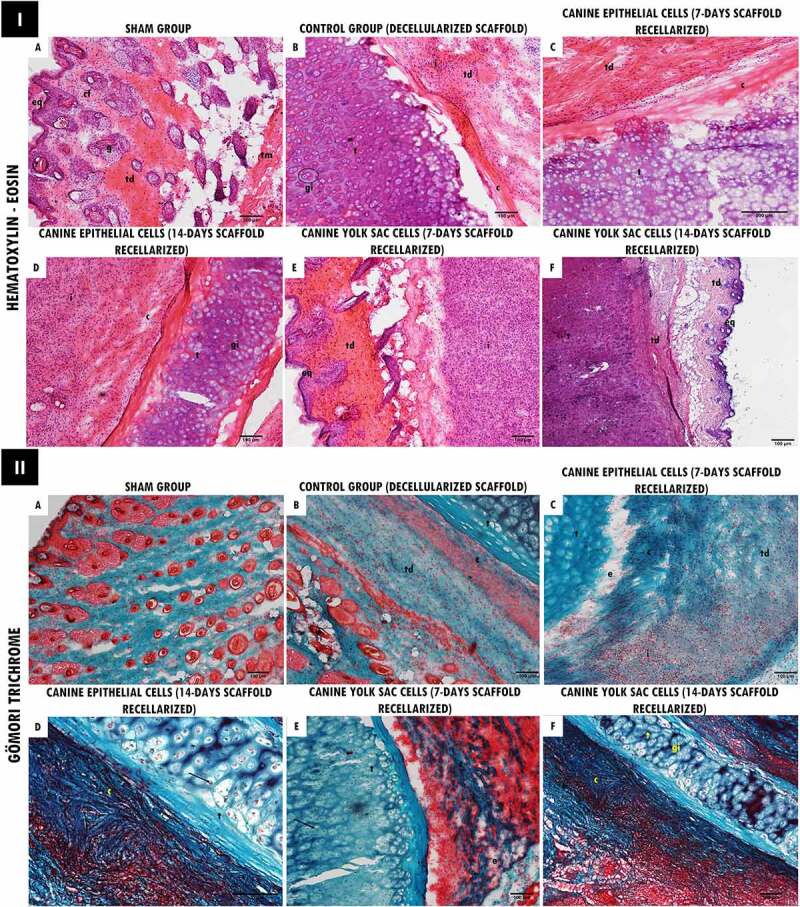
Histopathological analysis of decellularized and recellularized tracheal scaffolds (EpC/YS) implanted in the subcutaneous tissue of balb-C nude mice. I. H&E staining. In (a) Skin fragment: intact keratinized stratified squamous epithelium (eq), loose organized connective tissue (cf), sweat glands attached to the hair follicle (g), dense unmodeled connective tissue (td) and muscle tissue (tm). In (b-e) Deep dermis: area with the presence of implanted tracheal tissue (t), hyaline cartilage tissue, showing nucleated chondrocytes and formation of isogeny groups (gi) associated with ECM. Adjacent to this tissue, it is possible to observe a dense unmodeled connective tissue (td) associated with a discrete inflammatory infiltrate (i). Discrete presence of collagen fiber deposition surrounding the implanted tissue (c). In (f) Epidermis: intact keratinized stratified squamous epithelium (eq), dense unmodeled connective tissue (td). Deep dermis with an area showing a fragment of implanted tracheal tissue [hyaline cartilage] (t), chondrocytes and discrete formation of isogenous groups (gi). Adjacent to this tissue, it is possible to observe a dense unmodeled connective tissue (td) associated with a discrete mononuclear inflammatory infiltrate (i). Scale bar: (a-b; d-f: 100 µm, 10x/C: 500 µm, 20x). II. Gömori Trichrome stain. In (b) Area with the presence of implanted tracheal tissue (t), hyaline cartilage tissue, showing nucleated chondrocytes and formation of isogenous groups (gi) trapped by an extracellular matrix. Adjacent to this tissue it is possible to observe a dense unmodeled connective tissue (td). Discrete presence of collagen fiber deposition surrounding the implanted tissue (c). In (C) Deep dermis: area with the presence of implanted tracheal tissue (t) and region with mild edema (e). Adjacent to this tissue it is possible to observe a dense unmodeled connective tissue (td). Moderate presence of collagen fiber deposition surrounding the implanted tissue (c) and lymphoplasmacytic inflammatory infiltrate (i). In (DF) Area with presence of implanted tracheal tissue (t), hyaline cartilage tissue, presence of chondrocytes, highlighting the chondrocyte nucleus, emphasizing successful recellularization (arrow), in (e) discrete edema area (and) and in (f) presence of chondrocytes with visible nuclei and formation of isogeny groups (gi). Adjacent to this tissue, it is possible to observe a dense unmodeled connective tissue (td), deposition of collagen fibers surrounding the implanted tissue (c). Scale bar: (a- f: 100 µm, 10x).

Cell presence of in the chondrocyte/chondroblast gaps of the decellularized trachea with both cell types was observed by the H&E and Gömori Trichrome staining analysis (Figure 5). Depositions of collagen fibers surrounding the xenogenic cartilage were observed in the implanted area (Figures 5 I(c-d) and II(b-f)). In contrast, no fibrotic tissue process was observed in animals. The implanted cartilage contour and volume were preserved, and no severe inflammatory reactions were observed around the cartilage, and no chronic granulomatous inflammatory reaction after 45 days. By histological analysis, it was demonstrated focal regions of discrete inflammatory response in animals that received decellularized and recellularized scaffolds with both cell types for 7 days and 14 days (Figure 5 I(b,d-f)).

**Figure 6.**
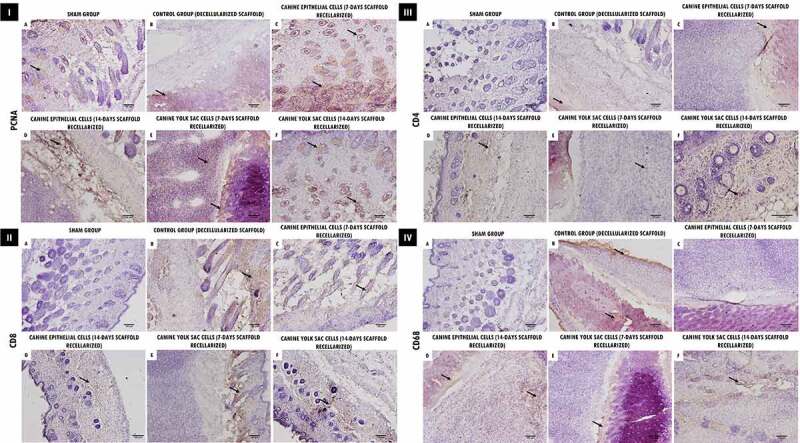
Immunohistochemical analysis of decellularized and recellularized tracheal scaffolds (EpC/YS) implanted in the subcutaneous tissue of balb-C nude mice. I. Proliferating cell nuclear antigen (PCNA). Observe proliferative activity in areas adjacent to the implant (arrows), demonstrating a positive interaction of scaffolds in the biocompatibility test. II. CD8. In (a, c and d) Note discrete positive marking for the presence of lymphocytes in areas adjacent to the implant (arrows), In (b, e and f) more intense marking for CD8 T cells, demonstrating an active role in the healing process of the implanted scaffolds in the biocompatibility test. III. CD4. Observe discrete positive marking for the presence of lymphocytes in areas adjacent to the implant (arrows), demonstrating an active role in the healing process of the implanted scaffolds in the biocompatibility test. IV. CD68. Observe more intense positive staining in control tissues (b), and in scaffolds recellularized at 14 days (d, f), respectively, demonstrating the presence of macrophages in areas adjacent to the implant (arrows), proving to be crucial to promote a remodeling response constructive use of the ECM. Scale Bar: (A-F: 100 µm, 10x).

Aiming to demonstrate the immune response and residual proliferation of the decellularized and recellularized scaffold implanted in the animals, we performed immunohistochemistry for PCNA, CD4, CD8, CD68. Proliferating cells (PCNA+) were observed adjacent to the scaffolds implantation site, as well as in the implanted trachea scaffold (Figure 6 I(a-f)). In comparison, the glycoproteins (CD4 and CD8) which play an essential role in the lymphocyte immune response, and as an MHC complex co-receptor (class type I and II), demonstrated a slight cellular infiltration expression of the adjacent implants regions (Figures 6 II and III(a-f)), as well as labeling in specific regions for CD68 in cell–ECM interactions, demonstrating macrophage activity in the analyzed tissues (Figure 6 IV(a-f)).

## 4. Discussion

Precursor studies conducted by KOJIMA et al., (2004) [25] targeting partial autologous transplantation of sheep tracheas demonstrated a survival rate of only 2–7 days after implantation, resulting from tracheomalacia and stenosis. Alternatively, synthetic tracheal prostheses covered by omental pedicle were transplanted in 20 dogs, and 13 animals died after 11 days of the procedure [26].

In this context, in order to advance efforts for partial trachea replacement, in this manuscript we aim to use different cell types for cell adhesion, proliferation, and migration in the decellularized ECM of the trachea. Although decellularized tracheal scaffolds can be applied in tissue engineering for allogeneic or xenogenic transplants, the reepithelialization process is necessary so that the combined treatment with immunosuppressants can give the patient longevity. Regarding this, the cell application is essential for long-term tissue outlast [5,14].

To achieve the partial reconstruction of the trachea, additionally to flexibility, the tissue requires a layer of epithelial cells lined with vascular tissue to mechanical traction and tube functionality of the in the physiological process of the organ and prevent tissue collapse [4,11,27]. Airway epithelial cells consist of a layer of pseudostratified cells that plays a central role in protecting the respiratory tract as a mechanical and immunological barrier [28]. Although such cells are implantation sources, tracheal cartilage can be recellularized with vascular progenitor cells and bone marrow mesenchymal cells, adipose tissue; however, there is no source of gold standard cells [29].

Our results demonstrate that it is possible to achieve decellularized canine tracheas with an optimized protocol, with tissue structures preserved, as well as the ECM proteins, crucial for the recellularization process. Moreover, canine decellularized trachea scaffolds achieve all the stringent requirements set forth in the literature that define how a decellularized scaffold should present itself [30]. Additionally, we isolated canine tracheal epithelial cells for cell culture with decellularized scaffolds. Associated with α-MEM culture medium, we supplemented with fetal bovine serum, which leads to better growth of tracheal epithelial cells [4,31,32].

In the present manuscript, the cells characterized by positive fluorescence for the markers suggest that we obtained an original cell population of epithelial cells derived from the canine trachea. Cell growth maintained its respective expression of epithelial marker (CK18) after seven days of culture. Furthermore, the cells showed a morphology changed after 24 hours and seven days, respectively. Our results corroborate those observed by [33]. The authors reveals that rat tracheal epithelial cells have positive expression of the CK18 marker, marking the intermediate filament of epithelial cells, as observed in our results. Additionally, the authors demonstrate VEGF positive expression when cultured *in vitro*, the same observed in EpC cells when recellularized with tracheal scaffolds.

Canine yolk sac cells, previously characterized [21], demonstrated proliferative and adhesive capacity when cultured for 7 and 14 days with tracheal scaffolds. The results obtained in our analyzes support the hypothesis that such cells are capable of being used in tissue engineering [22]. The growth factor VEGF is a multifunctional cytokine expressed in vascular cells, considered an important angiogenic mediator for cell differentiation and proliferation. *In vitro*, VEGF is able to promote the growth of vascular endothelial cells. In addition, both *in vitro* and *in vivo*, VEGF is also a survival factor for the respiratory tract endothelium, correlating epithelial/endothelial cells to induction of angiogenesis [34–36]. Studies conducted by [37] demonstrate VEGF high expression in granulation tissues when tracheal replacements are performed in children, demonstrating that the tissue cells themselves secrete the factor.

The epithelial cells proliferation and differentiation are one of the main characteristics of airway cells, as well as their ability to self-renew. Through the CK18 and β-Tubulin expression in the cytoskeleton (intermediate filaments) of epithelial cells, we demonstrate their differentiation ability. The immunolocalization of these markers was also observed in canine epithelial cells as cited by the authors [28,38,39] in mouse tracheal epithelial cells. These also expressed the transmembrane protein E-cadherin and N-cadherin, which interact through molecular bonds with catenins in cell-cell/cell-matrix adhesion [40]. The EpC cells culture and immunofluorescence characterization in this study can be associated with the results as already established for primary human airway epithelial cells [41,42] and mouse tracheal epithelial cells [28,43] that aim to investigate its role in the repair of respiratory diseases.

As observed by cells immunofluorescence attached to tracheal scaffolds, we identified by SEM that cells formed cell clusters in the tracheal tissue layers both in 7 days and 14 days. Our results are similar to the findings of ZANG *et al*., (2013) [11] which demonstrated that epithelial cells isolated from the rats trachea were susceptible to cell adhesion and growth in tracheal scaffolds by recellularization assay, and by [44] which demonstrated that epithelial and endothelial cells promote the re-epithelialization of the trachea when both cells were present, promoting the improvement of the lesion and maintaining vascularization.

Furthermore, recellularized scaffolds with EpC and YS cells demonstrated a proliferation when quantified by genomic DNA, as observed by the presence of cells, when compared to the results of decellularized samples [30]. Such results suggest the recellularization protocol success. In cases of short tracheal grafts (less than 5 cm), endothelial progenitor cells can grow inside the trachea to form blood vessels in order to keep the tissue active, since the low cell survival after implantation in vivo can generate tissue problems due to late neovascularization, as also noted by [29]. Also, the cell/ECM interaction may demonstrate proliferative action, since EpC cells modulate tissue composition, and associated with endothelial cells, such as YS, may promote airway vascular repair, as observed in studies conducted by [44–46]. *In vitro* analysis has shown satisfactory results to assess the performance of scaffolds, and *in vivo* implantation is often necessary.

Through the biocompatibility test, we aimed to evaluate the *in vivo* response of decellularized and recellularized tracheal fragments. Following, the main goal was to implant the scaffolds in mice to verify the immune response and whether they are reproducible for future preclinical trials. The results observed in the analyzes of the immune response and physiological behavior of our scaffolds support the success of the implantation, as observed in studies conducted by [16] that use decellularized tracheas for mice implantation. They demonstrated that the immune response was absent when cellular elements were removed from the ECM.

Further to these factors, our results demonstrated that cells associated with scaffolds can show a discrete inflammatory immune response when 7-days recellularized *in vitro*. In comparison, scaffolds recellularized for 14 days had a lower inflammatory response when applied *in vivo*. This could be associated due to the immune response action to the implanted scaffold, which can persist during the initial remodeling process and for several months after implantation [47,48]. Furthermore, the presence of macrophages is essential for promoting a constructive remodeling response, which can trigger an ‘adequate’ response in the tissue healing period, in addition to promoting a change in pro-inflammatory macrophages immediately after implantation for a population enriched in M2 macrophages (anti-inflammatory and pro-cure) for 7–14 days after implantation [48–51]. Besides in fact that our scaffolds *in vivo* implanted present discrete expression for macrophage markers: CD4, CD8 and CD68, and demonstrate that the tissue remained proliferative by the PCNA expression, as observed in precursor tissue implantation studies [1,52,53].

Finally, our initial reports demonstrate that recellularized scaffolds had cells adhered to ECM due to the preserved proteins after cell removing, maintaining their ability to adhere, proliferate, and differentiate adequately. Furthermore, our experiments proved to be reproducible; however, additional studies in large preclinical models are needed to validate the step before partial transplantation. The first step was taken.

## 5. Conclusion

Comparatively, natural EpC cells from the canine trachea showed greater adhesion and proliferation in decellularized scaffolds, demonstrating their ability to re-epithelialize the scaffold, which can provide functionality to the organ. On the other hand, YS cells demonstrated proliferative capacity and may be closely linked to the maintenance of tissue vascularization, preventing necrosis when used for clinical trials. Additionally, our scaffold has demonstrated the ability to remain active when implanted *in vivo*. With further enhancement, our approaches may be advantageous over conventional forms of recellularization and possible partial trachea replacement in tissue bioengineering applications.

## Supplementary Material

Supplemental Material

## Data Availability

All data generated or analyzed during this study are included in this article. Further enquiries can be directed to the corresponding author

## References

[1] Zang M, Zhang Q, Chang EI, et al. Decellularized tracheal matrix scaffold for tissue engineering. Plast Reconstructive Surg [Internet]. 2012;130:532–540. 10.1097/PRS.0b013e31825dc084 22929238

[2] Welman T, Michel S, Segaren N, et al. Bioengineering for organ transplantation: progress and challenges. Bioengineered [Internet]. 2015;6:257–261. [cited 2021 Dec 9]. Available from: https://www.tandfonline.com/doi/abs/10.1080/21655979.2015.1081320 26259720 10.1080/21655979.2015.1081320 PMC4825836

[3] Wright CD, Grillo HC, Wain JC, et al. Anastomotic complications after tracheal resection: prognostic factors and management. J Thorac Cardiovasc Surg [Internet]. 2004;128:731–739. Available from: https://linkinghub.elsevier.com/retrieve/pii/S0022522304009778 15514601 10.1016/j.jtcvs.2004.07.005

[4] Law JX, Liau LL, Aminuddin BS, et al. Tissue-engineered trachea: a review. Int J Pediatr Otorhinolaryngol [Internet]. 2016;91:55–63. [cited 2020 Jul 14]. Available from: https://linkinghub.elsevier.com/retrieve/pii/S0165587616303585 27863642 10.1016/j.ijporl.2016.10.012

[5] Delaere P, Van Raemdonck D. Tracheal replacement. J Thorac Dis. 2016;8:S186–96. 26981270 10.3978/j.issn.2072-1439.2016.01.85 PMC4775267

[6] Delaere PR, Vranckx JJ, Dooms C, et al. Tracheal autotransplantation: guidelines for optimal functional outcome. Laryngoscope. 2011;121(8):1708–1714. 21792958 10.1002/lary.21869

[7] Batioglu-Karaaltin A, Ovali E, Karaaltin MV, et al. Decellularization of trachea with combined techniques for tissue-engineered trachea transplantation. Clin Exp Otorhinolaryngol [Internet]. 2019;12(1):86–94. 30326701 10.21053/ceo.2018.00486 PMC6315211

[8] Grillo HC. Tracheal replacement: a critical review. Ann Thorac Surg [Internet]. 2002;73:1995–2004. [cited 2020 Apr 4]. Available from: https://linkinghub.elsevier.com/retrieve/pii/S0003497502035646 12078821 10.1016/s0003-4975(02)03564-6

[9] Vranckx JJ, Delaere P. The current status and outlook of trachea transplantation. Curr Opin Organ Transplant [Internet]. 2020;25: 601–608. Available from: https://journals.lww.com/10.1097/MOT.0000000000000808 33105201 10.1097/MOT.0000000000000808

[10] Conconi MT, De Coppi P, Di Liddo R, et al. Tracheal matrices, obtained by a detergent-enzymatic method, support in vitro the adhesion of chondrocytes and tracheal epithelial cells. Transplant Int [Internet]. 2005;18(6):727–734. [cited 2020 Jul 17]. Available from: https://onlinelibrary.wiley.com/doi/full/10.1111/j.1432-2277.2005.00082.x 10.1111/j.1432-2277.2005.00082.x 15910302

[11] Zang M, Zhang Q, Chang EI, et al. Decellularized tracheal matrix scaffold for tracheal tissue engineering. Plast Reconstr Surg [Internet]. 2013;132:549e–559e. [cited 2020 Jul 14]. Available from: https://pubmed.ncbi.nlm.nih.gov/24076702 10.1097/PRS.0b013e3182a013fc 24076702

[12] Etienne H, Fabre D, Gomez Caro A, et al. Tracheal replacement. Eur Respir J [Internet]. 2018;51: 1702211. Available from: http://erj.ersjournals.com/lookup/doi/10.1183/13993003.02211-2017 29444919 10.1183/13993003.02211-2017

[13] Delaere PR. Tracheal transplantation. Curr Opin Pulm Med [Internet]. 2012;18:313–320. [cited 2018 Dec 17]. Available from: http://www.ncbi.nlm.nih.gov/pubmed/22498734 22498734 10.1097/MCP.0b013e3283539673

[14] Delaere P, Van Raemdonck D, Vranckx J. Tracheal transplantation. Intensive Care Med [Internet]. 2019;45:391–393. 30430208 10.1007/s00134-018-5445-9

[15] Xu Y, Li D, Yin Z, et al. Tissue-engineered trachea regeneration using decellularized trachea matrix treated with laser micropore technique. Acta Biomater [Internet]. 2017;58:113–121. [cited 2020 Jul 21]. Available from: https://linkinghub.elsevier.com/retrieve/pii/S1742706117302908 28546133 10.1016/j.actbio.2017.05.010

[16] Kajbafzadeh AM, Sabetkish S, Sabetkish N, et al. In-vivo trachea regeneration: fabrication of a tissue-engineered trachea in nude mice using the body as a natural bioreactor. Surg Today [Internet]. 2015;45:1040–1048. [cited 2020 Jul 21]. Available from. https://link.springer.com/article/10.1007/s00595-014-0993-2 25062798 10.1007/s00595-014-0993-2

[17] Hong P, Bezuhly M, Graham ME, et al. Efficient decellularization of rabbit trachea to generate a tissue engineering scaffold biomatrix. Int J Pediatr Otorhinolaryngol [Internet]. 2018[cited 2020 Jul 17]; 112:67–74. Available from. https://pubmed.ncbi.nlm.nih.gov/30055743 30055743 10.1016/j.ijporl.2018.06.032

[18] Zhou Q, Ye X, Ran Q, et al. Trachea engineering using a centrifugation method and mouse-induced pluripotent stem cells. Tissue Eng Part C Methods [Internet]. 2018;24: 524–533. Available from: https://www.liebertpub.com/doi/10.1089/ten.tec.2018.0115 30101671 10.1089/ten.TEC.2018.0115

[19] Kojima K, Vacanti CA. Tissue engineering in the trachea. Anat Rec [Internet]. 2014;297:44–50. [cited 2020 Apr 4]. Available from: http://doi.wiley.com/10.1002/ar.22799 10.1002/ar.22799 24293389

[20] de Matias GSS, Rigoglio NN, Carreira ACO, et al. Optimization of canine placenta decellularization: an alternative source of biological scaffolds for regenerative medicine. Cells Tissues Organs [Internet]. 2018;205:217–225. [cited 2019 Apr 24]. Available from: https://www.karger.com/Article/FullText/492466 30199873 10.1159/000492466

[21] Fratini P, Carreira ACO, Alcântara D, et al. Endothelial differentiation of canine yolk sac cells transduced with VEGF. Res Vet Sci [Internet]. 2016;104:71–76. Available from: https://linkinghub.elsevier.com/retrieve/pii/S0034528815300977 26850540 10.1016/j.rvsc.2015.11.010

[22] Fratini P, Rigoglio NN, de Matias GSS, et al. Canine placenta recellularized using yolk sac cells with vascular endothelial growth factor. Biores Open Access [Internet]. 2018;7:101–106. [cited 2018 Nov 10]. Available from: http://www.ncbi.nlm.nih.gov/pubmed/30065855 30065855 10.1089/biores.2018.0014 PMC6056259

[23] de sá Schiavo Matias G, Batista VF, Carreira O, et al. Recellularization of canine placental extracellular matrix: mesenchymal stem cells applied to tissue bioengineering. J Stem Cells Res Dev Ther [Internet]. 2020;6:1–6. [cited 2021 Sep 21]. Available from: https://www.heraldopenaccess.us/openaccess/recellularization-of-canine-placental-extracellular-matrix-mesenchymal-stem-cells-applied-to-tissue-bioengineering

[24] Uemori T, Asada K, Kato I, et al. Amplification of the 16S-23S spacer region in rRNA operons of mycoplasmas by the polymerase chain reaction. Syst Appl Microbiol [Internet]. 1992;15:181–186. [cited 2018 Nov 28]. Available from: https://www.sciencedirect.com/science/article/abs/pii/S0723202011800895

[25] Kojima K, Ignotz RA, Kushibiki T, et al. Tissue-engineered trachea from sheep marrow stromal cells with transforming growth factor β2 released from biodegradable microspheres in a nude rat recipient. J Thorac Cardiovasc Surg [Internet]. 2004;128:147–153. Available from: https://linkinghub.elsevier.com/retrieve/pii/S0022522304004350 15224034 10.1016/j.jtcvs.2004.02.038

[26] Sekine T, Nakamura T, Liu Y, et al. Collagen coated Y-Shaped prosthesis for carinal replacement promotes regeneration of the tracheal epithelium. ASAIO J [Internet]. 2000;46:421–425. Available from: http://journals.lww.com/00002480-200007000-00010 10926138 10.1097/00002480-200007000-00010

[27] Weidenbecher M, Tucker HM, Gilpin DA, et al. Tissue-engineered trachea for airway reconstruction. Laryngoscope [Internet]. 2009;119:2118–2123. [cited 2020 Jul 17]. Available from. http://doi.wiley.com/10.1002/lary.20700 19806650 10.1002/lary.20700 PMC4811374

[28] Eenjes E, Mertens TCJ, Buscop-van Kempen MJ, et al. A novel method for expansion and differentiation of mouse tracheal epithelial cells in culture. Sci Rep [Internet]. 2018;8:7349. Available from: http://www.nature.com/articles/s41598-018-25799-6 29743551 10.1038/s41598-018-25799-6 PMC5943313

[29] Kaye R, Green GE, Smith LP. Tracheal replacement [internet]. In: Reference module in biomedical sciences. Elsevier; 2019. p. 281–284. [cited 2020 Jul 23]. Available from: https://linkinghub.elsevier.com/retrieve/pii/B9780128012383655484

[30] Crapo PM, Gilbert TW, Badylak SF. An overview of tissue and whole organ decellularization processes. Biomaterials [Internet]. 2011;32:3233–3243. Available from: https://linkinghub.elsevier.com/retrieve/pii/S0142961211000895 21296410 10.1016/j.biomaterials.2011.01.057 PMC3084613

[31] Chua KH, Aminuddin BS, Fuzina NH, et al. Human serum provided additional values in growth factors supplemented medium for human chondrocytes monolayer expansion and engineered cartilage construction. Med J Malaysia [Internet]. 2004;59(Suppl B:194–195. Available from: http://www.ncbi.nlm.nih.gov/pubmed/15468884 15468884

[32] Mohd Yunus MH, Chan Siang K, Hashim NI, et al. The effects of human serum to the morphology, proliferation and gene expression level of the respiratory epithelium in vitro. Tissue Cell [Internet]. 2014;46:233–240. [cited 2020 Jul 23]. Available from: https://pubmed.ncbi.nlm.nih.gov/24973262 24973262 10.1016/j.tice.2014.05.003

[33] Sun Z, Wang Y, Gong X, et al. Secretion of rat tracheal epithelial cells induces mesenchymal stem cells to differentiate into epithelial cells. Cell Biol Int [Internet]. 2012;36:169–175. [cited 2020 Jul 23]. Available from. https://onlinelibrary.wiley.com/doi/full/10.1042/CBI20110121 21919889 10.1042/CBI20110121

[34] Yang X-H, Man X-Y, Cai S-Q, et al. Expression of VEGFR-2 on HaCaT cells is regulated by VEGF and plays an active role in mediating VEGF induced effects. Biochem Biophys Res Commun [Internet]. 2006;349:31–38. [cited 2020 Jul 23]. Available from: https://pubmed.ncbi.nlm.nih.gov/16930552 16930552 10.1016/j.bbrc.2006.07.213

[35] Martin C, Coolen N, Wu Y, et al. CFTR dysfunction induces vascular endothelial growth factor synthesis in airway epithelium. Eur Respir J [Internet]. 2013;42:1553–1562. Available from: http://erj.ersjournals.com/lookup/doi/10.1183/09031936.00164212 23520314 10.1183/09031936.00164212

[36] Hines EA, Sun X. Tissue crosstalk in lung development. J Cell Biochem [Internet]. 2014;115: 1469–1477. Available from: http://doi.wiley.com/10.1002/jcb.24811 24644090 10.1002/jcb.24811 PMC8631609

[37] Pokharel RP, Maeda K, Yamamoto T, et al. Expression of vascular endothelial growth factor in exuberant tracheal granulation tissue in children. J Pathol [Internet]. 1999;188:82–86. [cited 2020 Jul 23]. Available from: https://onlinelibrary.wiley.com/doi/full/10.1002/%28SICI%291096-9896%28199905%29188%3A1%3C82%3A%3AAID-PATH324%3E3.0.CO%3B2-4 10398145 10.1002/(SICI)1096-9896(199905)188:1<82::AID-PATH324>3.0.CO;2-4

[38] You Y, Richer EJ, Huang T, et al. Growth and differentiation of mouse tracheal epithelial cells: selection of a proliferative population. Am J Physiol Lung Cell Mol Physiol [Internet]. 2002;283:L1315–21. [cited 2020 Jul 23]. Available from: www.ajplung.org 12388377 10.1152/ajplung.00169.2002

[39] Ikeda M, Imaizumi M, Yoshie S, et al., Implantation of induced pluripotent stem cell–derived tracheal epithelial cells. Ann Otol Rhinol Laryngol [Internet]. 2017;126:517–524. 28604083 10.1177/0003489417713504

[40] Walimbe T, Panitch A, Sivasankar MP. An in vitro scaffold-free epithelial-fibroblast coculture model for the larynx. Laryngoscope [Internet]. 2017;127:E185–92. [cited 2020 Jul 23]. Available from: http://doi.wiley.com/10.1002/lary.26388 27859361 10.1002/lary.26388 PMC5465635

[41] Schilders KAA, Eenjes E, van Riet S, et al. Regeneration of the lung: lung stem cells and the development of lung mimicking devices. Respir Res [Internet]. 2016;17:44. [cited 2020 Jul 23]. Available from: https://pubmed.ncbi.nlm.nih.gov/27107715 27107715 10.1186/s12931-016-0358-z PMC4842297

[42] Nichols JE, Niles JA, Vega Sp, et al. Modeling the lung: design and development of tissue engineered macro- and micro-physiologic lung models for research use. Exp Biol Med [Internet]. 2014;239:1135–1169. [cited 2020 Jul 23]. Available from: https://pubmed.ncbi.nlm.nih.gov/24962174 10.1177/1535370214536679 24962174

[43] Rock JR, Randell SH, Hogan BLM. Airway basal stem cells: a perspective on their roles in epithelial homeostasis and remodeling. Dis Model Mech [Internet]. 2010;3:545–556. 20699479 10.1242/dmm.006031 PMC2931533

[44] Zani BG, Kojima K, Vacanti CA, et al. Tissue-engineered endothelial and epithelial implants differentially and synergistically regulate airway repair. Proc Nat Acad Sci [Internet]. 2008;105:7046–7051. [cited 2020 Jul 23]. Available from: www.pnas.orgcgidoi10.1073pnas.0802463105 18458330 10.1073/pnas.0802463105 PMC2383974

[45] Methe H, Groothuis A, Sayegh MH, et al. Matrix adherence of endothelial cells attenuates immune reactivity: induction of hyporesponsiveness in allo‐ and xenogeneic models. FASEB J [Internet]. 2007;21: 1515–1526. Available from https://onlinelibrary.wiley.com/doi/abs/10.1096/fj.06-7051com 17264166 10.1096/fj.06-7051com

[46] Methe H, Edelman ER. Tissue engineering of endothelial cells and the immune response. Transplant Proc [Internet]. 2006;38:3293–3299. Available from: https://linkinghub.elsevier.com/retrieve/pii/S0041134506012814 17175253 10.1016/j.transproceed.2006.10.052 PMC1865503

[47] Anderson JM. Inflammatory Response to Implants. ASAIO J [Internet]. 1988;34:101–107. [cited 2021 Jul 13]. Available from: https://pubmed.ncbi.nlm.nih.gov/3285869 10.1097/00002480-198804000-00005 3285869

[48] Brown BN, Badylak SF. Extracellular matrix as an inductive scaffold for functional tissue reconstruction. In: Translating regenerative medicine to the clinic. 2015;163(4):268–285. 10.1016/j.trsl.2013.11.003. PMC4203714 24291155

[49] Brown BN, Badylak SF. Extracellular matrix as an inductive scaffold for functional tissue reconstruction. Transl Res [Internet]. 2014;163:268–285. [cited 2021 Jul 13]. Available from: pmc/articles/PMC4203714 24291155 10.1016/j.trsl.2013.11.003 PMC4203714

[50] Brown BN, Londono R, Tottey S, et al. Macrophage phenotype as a predictor of constructive remodeling following the implantation of biologically derived surgical mesh materials. Acta Biomater [Internet]. 2012;8:978–987. [cited 2021 Jul 13]. Available from: pmc/articles/PMC4325370 22166681 10.1016/j.actbio.2011.11.031 PMC4325370

[51] Badylak SF, Valentin JE, Ravindra AK, et al. Macrophage phenotype as a determinant of biologic scaffold remodeling. Tissue Eng Part A [Internet]. 2008; 14:1835–1842. 18950271 10.1089/ten.tea.2007.0264

[52] Carvalho CMF, Leonel LCPC, Cañada RR, et al. Comparison between placental and skeletal muscle ECM: in vivo implantation. Connect Tissue Res [Internet]. 2020;1–14.cited 2021 May 12. Available from: https://pubmed.ncbi.nlm.nih.gov/33106052 33106052 10.1080/03008207.2020.1834540

[53] Alberti KA, Xu Q. Biocompatibility and degradation of tendon-derived scaffolds. Regen Biomater [Internet]. 2016;3:1–11. [cited 2021 Jul 16]. Available from: pmc/articles/PMC4723279 26816651 10.1093/rb/rbv023 PMC4723279

