# Supplementary figures and images for: In vivo biocompatibility analysis of the recellularized canine tracheal scaffolds with canine epithelial and endothelial progenitor cells
Source: Bioengineered. 2022 Feb 2;13(2):3551–65. doi: 10.1080/21655979.2021.2020392 (PMC8974223; doi:10.1080/21655979.2021.2020392)

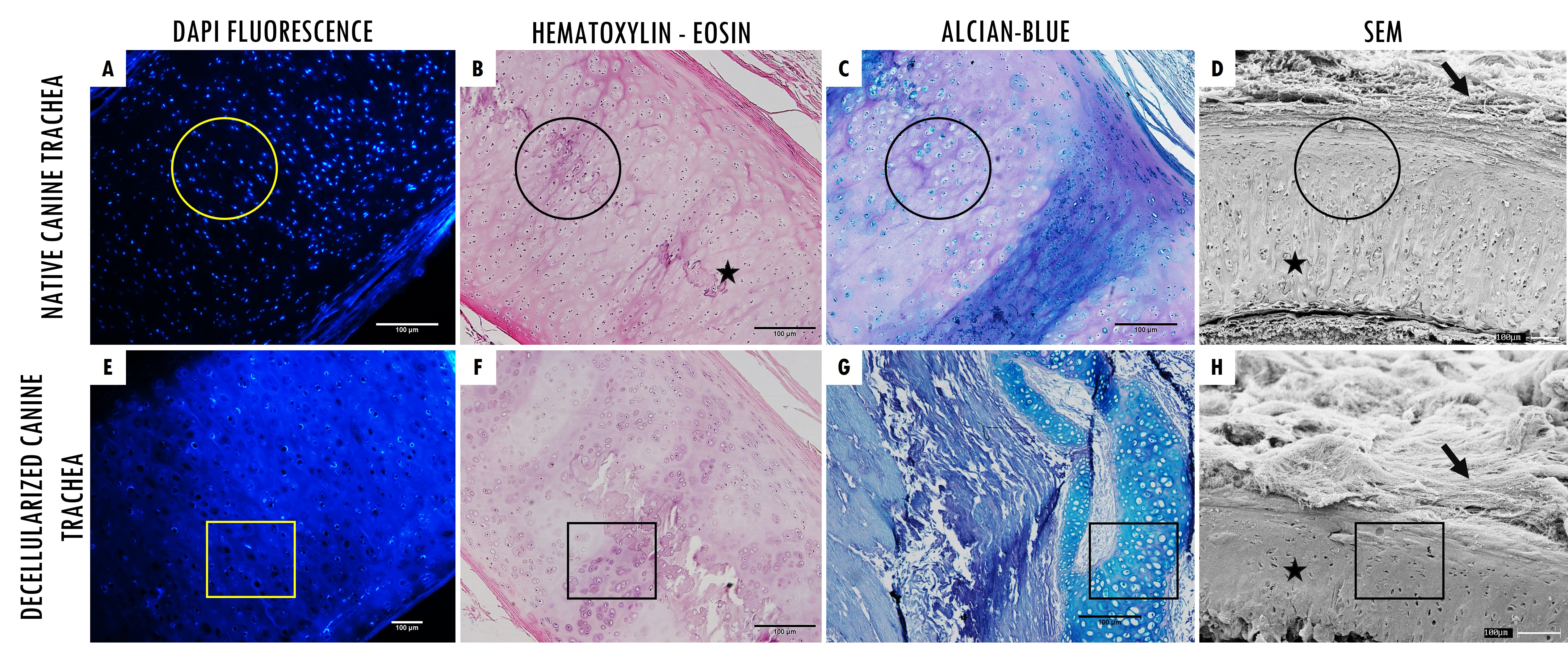

Supplement: Supplemental Material [file KBIE_A_2020392_SM0523.zip › supplementary/Suppl Fig 1.tiff]

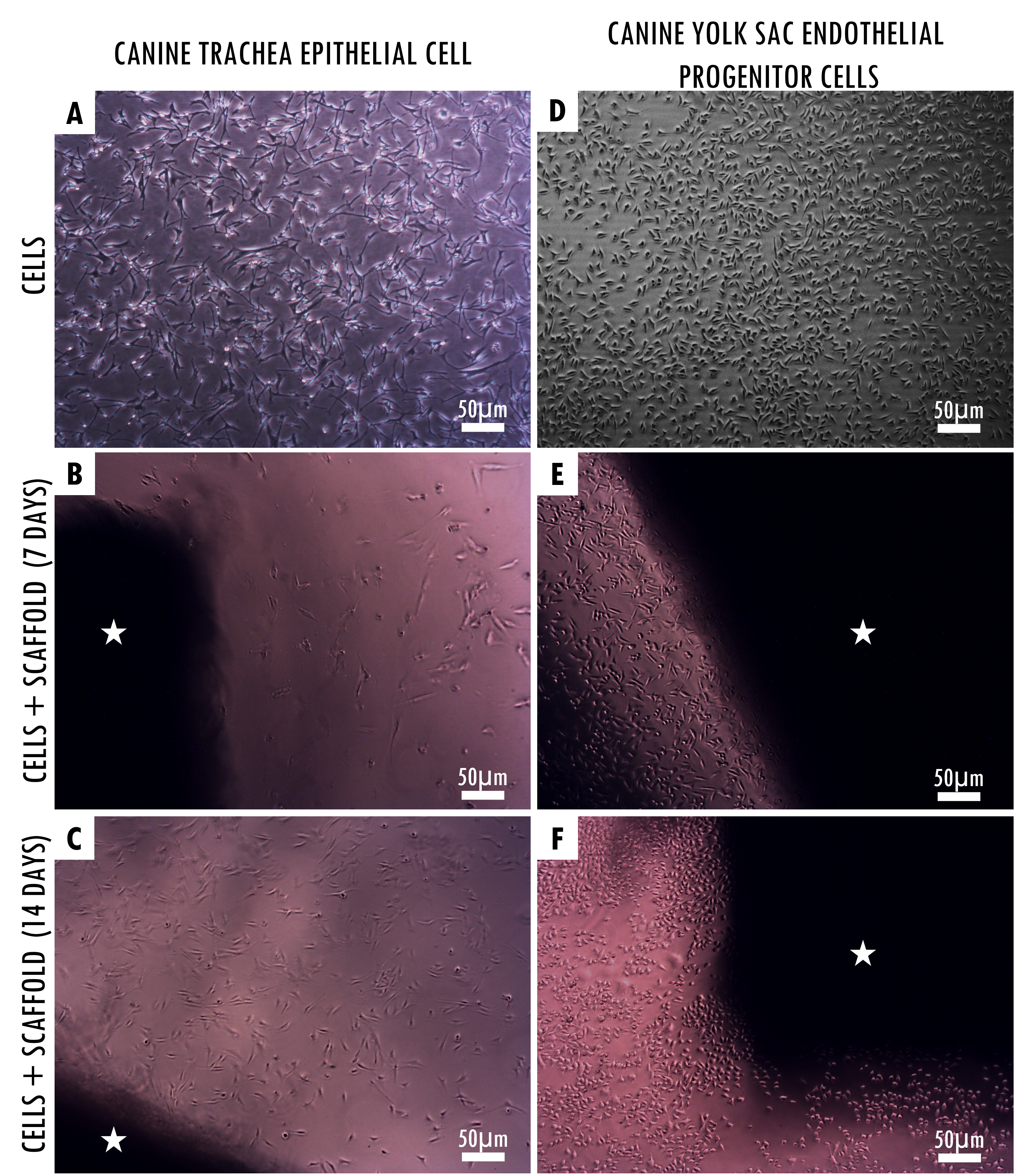

Supplement: Supplemental Material [file KBIE_A_2020392_SM0523.zip › supplementary/Suppl Fig 2.tiff]

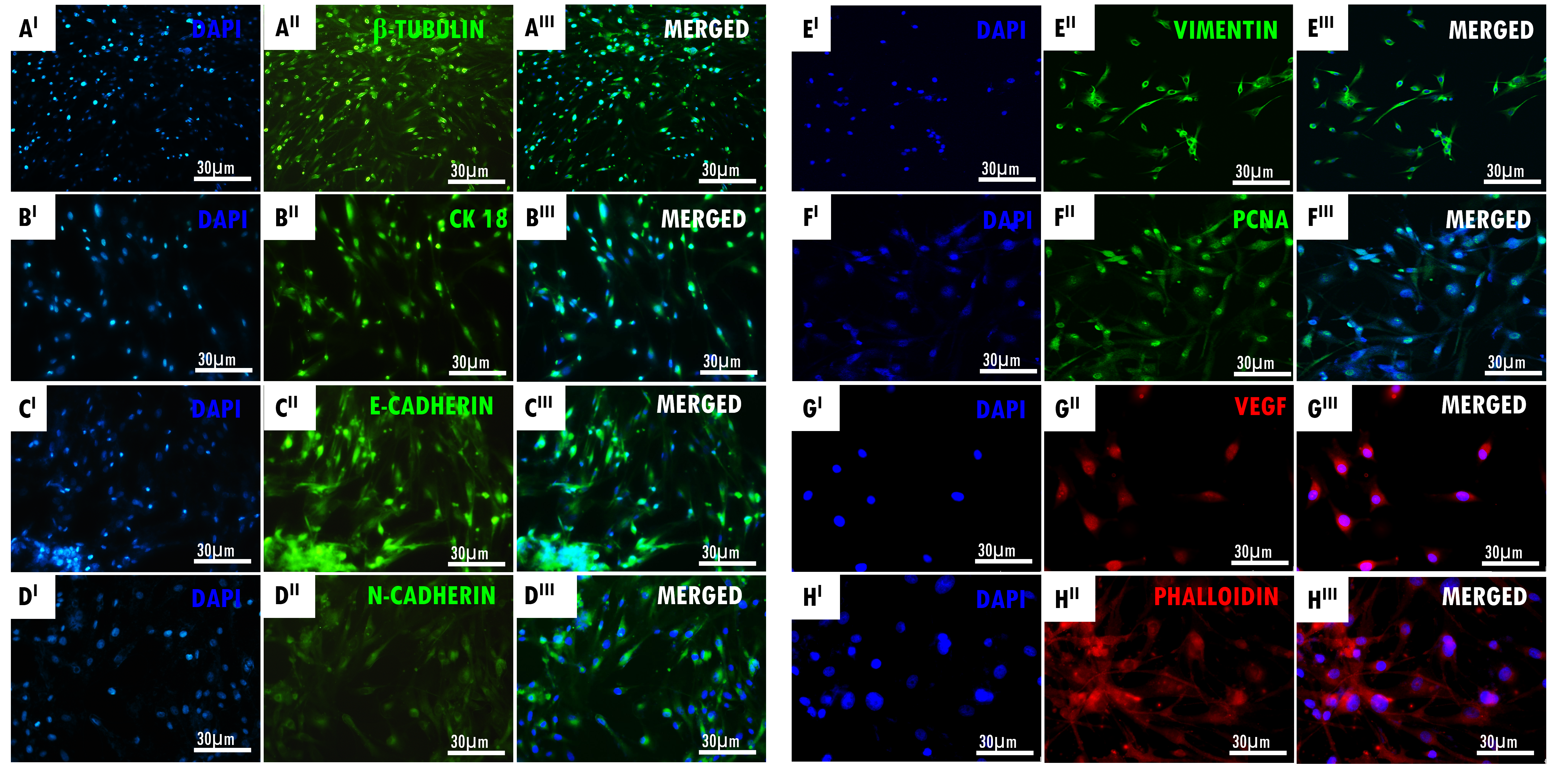

Supplement: Supplemental Material [file KBIE_A_2020392_SM0523.zip › supplementary/Suppl Fig 3.tiff]

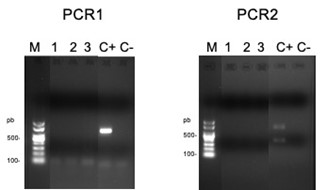

Supplement: Supplemental Material [file KBIE_A_2020392_SM0523.zip › supplementary/Suppl Fig 4.tiff]
